# Predictors of long-term outcome of first-episode schizophrenia: A ten-year follow-up study

**DOI:** 10.4103/0019-5545.74306

**Published:** 2010

**Authors:** Amresh Shrivastava, Nilesh Shah, Megan Johnston, Larry Stitt, Meghana Thakar

**Affiliations:** Regional Mental Health Care, 467 Sunset Drive, St. Thomas, Ontario, Canada N5H 3V; 1 Mental Health Foundation of India (PRERANA Charitable Trust) and Silver Mind Hospital, 209 Shivkripa Complex, Thane, Mumbai, Maharashtra 400 602, India; 2 Department of Psychology, University of Toronto, 100 St. George St., Toronto, Ontario, Canada, M5S 3G3; 3 Department of Epidemiology & Biostatistics, Schulich School of Medicine & Dentistry, The University of Western Ontario, London, Ontario, N6A 5C1; 4 LTMG Hospital, University of Mumbai, Sion, Mumbai, Maharashtra 400 022, India

**Keywords:** Follow-up studies, outcome assessment, psychosocial factors, schizophrenia, treatment outcome

## Abstract

**Objective:**

Schizophrenia is a severe mental disorder for which final outcomes continue to be unfavorable. The main objectives of this research were to examine and determine the baseline predictors of outcome status of first-episode schizophrenia in a long-term follow-up of ten years and of recovery ten years later.

**Materials and Methods:**

The study was carried out in a non-governmental, psychiatric hospital and participants consisted of patients available for assessment ten years following their initial diagnosis. Outcome was assessed on clinical and social parameters. Clinical measures of outcome included psychopathology, hospitalization, and suicidality. Social parameters included quality of life functioning, employability, interpersonal functioning, and the ability to live independently.

**Results:**

In our sample, mean positive symptoms’ score were reduced by more than 65% between baseline and endpoint. The percentage of reduction in scores of negative symptoms is much less than reduction in positive symptoms. It was observed that only 23-25% patients showed social recovery on two or three different parameters. Additionally, fewer negative symptoms, lower depression scores, and low levels of aggression at baseline predicted good outcome. A higher level of positive symptoms at baseline also predicted recovery. The two social variables that predicted later outcomes were initially high levels of work performance and the ability to live independently at baseline.

**Conclusions:**

Clinical information is not sufficient to make an accurate prediction of outcome status; rather, outcome depends upon multiple factors (including social parameters). A major implication of this research is the argument for moving toward a comprehensive assessment of outcome and to plan management accordingly. Bringing social outcome measures to the forefront and into the communities will allow for a more patient-centric approach. It also opens newer vistas for addressing the complex interaction of clinical and social parameters.

## INTRODUCTION

Schizophrenia is a severe mental disorder, most commonly, though not always, affecting young individuals at the time when the personality is growing and cognitive faculties are maturing. Although there have been some advancements in treatment, final outcomes in the management of schizophrenia continue to be unfavorable. Jobe and Harrow[1] observed that although a great deal of variance in outcome occurred across many studies, schizophrenia is nevertheless a disorder with relatively poor outcome. Patients with schizophrenia consistently showed a poorer course of illness and poorer outcomes than patients with other psychotic and non-psychotic psychiatric disorders. In general, the outcome remains almost the same even where intervention has been done during the first episode.[2,3] Further, long-term studies of schizophrenia do not provide a more promising picture.

A large study by Helgason[4] demonstrated that the majority of schizophrenia patients experienced serious difficulties in achieving a satisfactory quality of life. Over half of them never married, 32% of those who married had divorced, and a similar number had lost the support of their families. Patients who underwent treatment improved but only 29% achieved an acceptable level of health. Such figures point to the fact that changes are required in basic treatment structure if this situation is to be altered. In the heterogeneous group of schizophrenia syndromes, identifying subgroups with good outcome remains a challenge. The long-term outcome of schizophrenia should address two main questions: what we can do to maximize outcome and achieve complete social integration and when; and how we can identify subjects who may not respond favorably to treatments to facilitate planning for impending disability right from the beginning.

The present study was conducted in Mumbai city, the fifth most populous metro in the world. Patients and their families here lead a challenging and stressful city life. It is not very well known if schizophrenia outcome is in any way influenced by the urban culture of the city. Determinants of outcome have been repeatedly investigated and it has been argued that schizophrenia is a heterogeneous disorder, which has variable outcomes across the world.[5] Cross-national research by the World Health Organization[6,7] in particular has led to the belief that schizophrenia has better course and outcome in developing countries. Long-term outcome in developing countries, eastern countries, and non-industrialized countries, particularly in India has been reported to be good and favorable, which may translate into a range of 22–75% of individuals demonstrating good outcome.[8–10] The Madras longitudinal study from India reported a good outcome as high as 75% with significant numbers engaging in employment 10 to 15 years later.[11] The hypothesis proposed to explain this better outcome in a developing country versus Western countries is interesting. Some of the factors proposed to have a role in this outcome are family interaction, family pathology, expressed emotions, cultural factors, genetics and the nature of illness itself.[11]

However, this cultural difference in outcome has been challenged recently and it is argued that there has been a selection bias against the most severe cases in the studies from developing countries.[5,8] Further, outcome studies of schizophrenia in India have shown unusually high mortality rates in this group.[12] A 20-year prospective study of individuals with schizophrenia in India demonstrated that although a decline in symptoms was evidenced, the predominant pattern was that of repeated episodes of psychosis not necessarily followed by remission.[11]

As more research continues to argue against better outcome in developing countries, a reconsideration of this common belief seems imperative.[13] This new perspective on cross-national outcomes is consistent with the biological theory of schizophrenia present in most models of this disease.[14–16] in other words, schizophrenia is a biological disease and is universal in terms of its outcome. Several studies of illness course and outcome have demonstrated that across cultures a young age, the male gender, preexisting cognitive dysfunction, poor psychosocial support, and high levels of expressed emotions are strongly correlated to poor outcome.[17]

The main objectives of this research were to examine the outcome status of first-episode schizophrenia in a long-term follow-up and comment upon the appropriateness of a multidimensional approach to the measurement of outcome, at least on two different parameters: clinical and social. Previous research has highlighted the importance of measuring outcome using a multidimensional approach.[18] The present study examined the following questions in a cohort of first-time-hospitalized patients: What is the status of a long-term comprehensive measure of outcome, i.e. clinical recovery as well as social recovery? What are the predictors of ten-year outcomes?

## MATERIALS AND METHODS

### Setting

The study was carried out in a non-governmental, psychiatric hospital certified as a psychiatric facility by the State Government as per the Indian Mental Health Act 1987. An Independent Ethics Commission approved the study.

### Subjects

A total of 200 patients hospitalized for first-episode schizophrenia were recruited and provided consent. The mean age of this sample was 28.8 years (*SD*=8.2; range 17-47) and 74 patients (73.3%) were male. Recruited participants were consecutive admissions who met the inclusion and exclusion criteria. The inclusion criteria for participants of the study were: first hospitalization, confirmed diagnosis as per diagnostic and statistical manual of mental disorders (DSM III), consistent treatment and follow-up for ten years, and informed consent for assessment. The exclusion criteria for potential study participants were: substance abuse, outpatients or non-hospitalized individuals, evidence of significant previous treatment (administration of antipsychotics for a period exceeding two weeks prior to hospitalization), significant medical or neurological illness, and an absence of objective information from the key relatives.

We recruited 200 first-episode schizophrenic patients, out of which 101 were available at the end point for assessment. Six patients showed a changed diagnosis and hence were excluded from the assessment. During the course of follow-up, 24 patients changed the treatment setting, 12 migrated out of the city, 15 were admitted to a long-term facility, four patients died (two from natural causes and two from suicide), and 38 were “lost in follow-up” due to discontinuation, poor compliance, lack of consistency, and poor follow-up. A comparison of those who followed up and those who did not was not studied as it was beyond the scope and objective of the present research. Thus, those lost to follow-up were mainly a result of non-illness and non-therapeutic factors.

All subjects had untreated first-episode schizophrenia but not strictly drug-naïve due to prescribing practices. The consenting participants were recruited as per criteria for inclusion and exclusion. All patients were screened at intake for a diagnosis of schizophrenia as per DSM–III. Only those qualifying for non-affective psychosis were included. Recruited participants were assessed in detail using psychiatric measurement tools for psychopathology, its nature and severity, and functional status. Ten years post treatment the available patients were reassessed. Their diagnosis was reconfirmed as per DSM-IV. These patients were assessed for outcome using the Clinical Global Impression Scale-Improvement (CGIS –I).

At the 10-year follow-up point, 101 patients were available and these comprised the participants in the present sample. At the endpoint, the mean age was 39.2 years (SD=7.9; range 22-58). All participants had a minimum of Grade 12 education, living in catchments with families, and belonging to the middle-class socioeconomic group. The majority of participants were hospitalized in an emergency.

### Assessment Parameters and Tools

Measures of outcome on multidimensional criteria were divided into clinical and social recovery. Clinical parameters consisted of symptoms, psychopathology, side-effects, depression, aggression, hospitalization, and suicidality. Social parameters were measured items which identified the level of quality of life functioning, employability, return to education, interpersonal functioning, level of family burden cause, and the ability to live independently and were measured by locally structured scales. Outcome was measured using the Clinical Global Impression Scale (CGIS).[19,20]

#### Clinical outcome

Clinical outcome was measured by: 1) psychopathology (positive symptoms, negative symptoms and disorganization) using the Positive and Negative Syndromes Scale (PANSS);[21] 2) extra-pyramidal function assessed with the Abnormal Involuntary Movement Scale (AIMS); (scores greater than 2 taken as significant);[22] and 3) depressive symptoms,[23] aggression, hospitalization and suicidality measured on a scale of 1-5.

#### Social outcome

Social outcome was measured using the following measurement tools: 1) Quality of Life (QOL);[24] 2) Global Assessment of Functioning (GAF); 3) independent living; 4) interpersonal social function; 5) work–school function; 6)family burden; and 7) social burden. The last five variables were assessed using a 1-5 scale, 1 being the poorest and 5 being the highest positive outcome. This semi--structured scale has been tested in local conditions and used in other studies.[25]

#### Overall outcome criteria

The primary outcome measure was a score of 2 or less on the CGIS-Improvement subscale (improved or much improved). The secondary criteria for “good outcome” were: a patient showing good compliance (less than 80%), not being hospitalized for a minimum of two preceding years, GAF score greater than 80, QOL score greater than 80, AIMS score less than 2, scores greater than 3 on scales of social functions, independent living, education, and social burden (reverse scored).

### Follow-up assessment

The follow-up assessment was performed at the end of the completed 10 years of treatment. Good outcome was defined as those showing ‘improvement’ and ‘much improvement’ on the CGIS. Clinical assessment was administered by two experienced clinicians. Psychometrics were performed by a clinical psychologist. Also, at the follow-up, diagnosis was reconfirmed using the DSM-IV-TR.[26]

### Statistical analysis

Descriptive statistics for demographic characteristics and patient scores at baseline and after 10 years of follow-up were calculated. To test the degree of change between baseline and final follow-up assessments, paired *t*-tests were used for continuous variables and McNemar’s chi-square tests were used for dichotomous variables. Logistic regression was used to evaluate univariable associations between baseline characteristics and recovery as defined by the CGIS. Those associations found to be significant at the 0.10 levels were entered into a stepwise logistic model, allowing for entry at the 0.05 levels. Lack of fit was evaluated using the Hosmer-Lemeshow goodness of fit statistic.

The seven clinical parameters including negative symptoms, positive symptoms, disorganization, hospitalization in the past two years, suicidality, extra-pyramidal function, and aggression at the 10-year follow-up were categorized as normal or abnormal and the number of normal parameters was determined. Similarly, the five social parameters of independent living, interpersonal/social, work/school, quality of life, and GAF at the 10-year follow-up were categorized as normal or abnormal and the number of normal parameters was determined. The number of normal clinical parameters and the number of normal social parameters for those who recovered according to the CGIS were compared to those who did not recover using a Wilcoxon two-sample test. The relationship between the number of normal clinical parameters observed and the number of normal social parameters observed was evaluated using a Spearman rank correlation coefficient. The data was analyzed using SAS Version 9.1. *P* values less than 0.05 were considered to be statistically significant.

## RESULTS

The results revealed that 61 out of 101 patients showed ‘improved’ and a ‘much improved’ state on CGIS. Of the sample assessed at the 10-year follow-up, 72% of participants were treated regularly and 21% were treated occasionally depending on their needs. Thirty-six percent of the patients were never hospitalized after index hospitalization, while 63% of the patients were repeatedly hospitalized (27% with only one or two admissions during the 10-year period).

### Comparison of baseline to follow-up

#### Baseline assessment

Reasons for hospitalization included episodes of violence, uncontrollable aggression, attempted suicide and suicidal crisis. For the 101 patients that completed the follow-up, the mean age at intake was 28.8 (SD=8.2) years and the mean illness duration was 12.7 (SD=7.3) months. These patients had severe psychopathology and scored high on the total PANSS score, the positive score, the negative score, and the depressive symptoms score [Table 1]. Sixty-four patients (63.4%) demonstrated significant aggression. Their global functioning was poor (M=48.3, SD=11.0). The severity of illness averaged out to 5.5 (SD=0.9) on CGIS-S. Seventy-three patients (72.3%) had experienced suicidal thoughts, gestures or plans. On social parameters, 75 patients (74.3%) did not demonstrate satisfactory interpersonal functioning, 26 patients (25.7%) were employed or engaged in productive work, and 89 subjects (88.1%) completely lacked the ability to live independently. Interestingly, however, significant family burden was observed in only four patients (4.0%). Five patients (5.0%) showed significant extra-pyramidal symptoms at baseline, possibly because of neuroleptic exposure in the community prior to hospitalization.

**Table 1:** Comparison of outcomes between baseline and follow-up

	Means (SD)		Paired
	Baseline (%)	Follow-up (%)	Comparison
Outcome			
PANSS†	106.0 (13.9)	51.6 (8.9)	t_100_=38.04, *P*<.001
Positive symptoms	28.3 (5.1)	8.7 (3.9)	t_100_=29.53, *P*<.001
Negative symptoms	23.5 (6.9)	12.2 (7.4)	t_100_=12.27, *P*<.001
GP‡	54.3 (16.8)	29.1 (11.9)	t_100_=13.82, *P*<.001
HDRS§	17.5 (6.1)	13.1 (5.2)	t_96_=6.13, *P*<.001
GAF¶	48.3 (11.0)	78.9 (11.7)	t_95_=22.57, *P*<.001
Outcome (Cutoff)			
Disorganization (>3)	88 (87.1)	40 (39.6)	*χ*^2^=37.23, *P*<.001
IP SocialΦ (<3)	75 (74.3)	73 (72.3)	*χ*^2^=0.090, P=.768
Work (<3)	74 (73.3)	75 (74.3)	*χ*^2^=0.040, P=.842
AIMSΨ (>2)	5 (5.0)	35 (34.7)	*χ*^2^=25.00, *P*<.001
Disturbed living (<3)	89 (88.1)	48 (47.5)	*χ*^2^=34.38, *P*<.001
Aggression (>2)	64 (63.4)	39 (38.6)	*χ*^2^=10.59, P=.001
Family burden (>3)	4 (4.0)	54 (53.5)	*χ*^2^=50.00, *P*<.001
Suicidality (>1)	73 (72.3)	51 (50.5)	*χ*^2^=9.68, *P*<.001

PANSS^†^ - Positive and negative syndrome scale;

GP^‡^ - General psychopathology subscale of PANSS;

HDRS^§^ - Hamilton depression rating scale;

GAF^¶^ - Global assessment of functioning;

IP Social^Φ^ - Interpersonal/ Social;

AIMS^Ψ^ - Abnormal involuntary movement scale;

Frequency with Abnormal Scores (%)

#### Endpoint assessment

At the endpoint of the 10-year follow-up, statistically significant decreases were found in total PANSS, positive score, negative score, general psychopathology, disorganization, and scores on [Table 1]. Statistically significant reductions in suicidality and aggression were observed. Global functioning showed significant improvement. Although more patients were able to live independently, neither interpersonal functioning nor employment changed significantly. Seventy-four percent of participants were unemployed at baseline and 75% remained unemployed at the end of 10 years’ treatment. Onset of illness is related to unemployment but long-term treatment also did not provide any increase in the employment rate. However, significantly more patients developed extra-pyramidal symptoms and were a burden on their families.

#### Comparison of baseline to follow-up

Overall, when examining all individuals available at the follow-up assessment, *t*-tests showed significant decreases in all clinical symptoms (positive, negative, depressive, etc.; all *P*s<.001). Additionally, between baseline and follow-up there was a significant decrease in the number of patients unable to live independently (*P*<.001) and decrease in patients who were a burden to their families (*P*<.001). However, no significant changes were seen in work performance and interpersonal skills during the 10 years between assessments [Table 1].

### Determinants of good outcome

Longer symptom duration, later age at the intake, higher total PANNS score, greater positive symptoms, fewer negative symptoms, higher general psychopathology scores, lower scores on HDRS, positive employment status and independent living, and fewer symptoms of aggression were significantly associated with recovery as good outcome [Table 2]. In a stepwise multivariable logistic regression, age at onset, PANNS, positive symptoms, negative symptoms, general psychopathology score of PANSS, HDRS score, employment status, independent living, and symptoms of aggression were found to be independently associated with recovery.

**Table 2:** Comparison of recovered and non-recovered patients at follow-up

Outcome	Non-recovered Means (SD)	Recovered Means (SD)	Difference Means (SD)	95% CI	P Valueλ
Age	33.4 (7.2)	41.8 (7.3)	-6.5 (7.3)	-9.4, -3.5	<.001
PANSS†	54.9 (9.0)	49.4 (8.2)	5.5 (8.5)	2.1, 9.0	.002
Pos. symptoms	9.8 (3.8)	8.0 (3.9)	1.9 (3.8)	.3, 3.4	.019
Neg. symptoms	15.4 (6.0)	10.1 (7.5)	5.4 (6.9)	2.6, 8.2	<.001
GP‡	26.3 (10.0)	31.0 (12.7)	-4.7 (11.7)	-9.4, 0.1	.053
HDRS§	14.1 (4.9)	12.5 (5.3	1.6 (5.2)	-0.5, 3.7	.141
GAF¶	19.9 (10.7)	78.3 (12.2)	1.6 (11.7)	-3.3, 6.5	.523
QOLΦ	54.5 (7.5)	76.2 (11.5)	-21.7 (10.1)	-25.8, -17.6	<.001

PANSS^†^ - Positive and negative syndrome scale;

GP^‡^ - General psychopathology subscale of PANSS;

HDRS^§^ - Hamilton depression rating scale;

GAF^¶^ - Global assessment of functioning;

QOL^Φ^ - Quality of life;

λ Statistical comparisons obtained using unpaired *t*–tests.

For QOL there was evidence that the variances are not equal. However, via a non-parametric test (Wilcoxon two-sample test) a between-group statistically significant difference was observed (*P*<.001)

#### Comparison between recovered and non-recovered

There was a statistically significant difference in the symptoms shown by the recovered (good outcome) and non-recovered (poor outcome) groups at baseline, in terms of both positive (mean difference=1.9, *P*=.019) and negative symptoms (mean difference=5.4, *P*<.001). The two groups also differed significantly on the parameters of interpersonal-social functioning (*P*=.001) and quality of life (*P*<.001). At baseline, the two groups (recovered and non-recovered) did not differ in the presence of extra-pyramidal symptoms, aggression, family burden, level of suicidality or general level of functioning. Additionally, the scores of total clinical symptoms at baseline and gender did not predict good or poor outcome (Table 2 for all comparisons between recovered and non-recovered groups).

The recovered and non-recovered groups also differed significantly at the 10-year follow-up on the parameters of repeated hospitalization, presence of positive symptoms and presence of negative symptoms. Non-recovered patients had more frequent repeated hospitalizations (92.5% of non-recovered patients), more frequent positive symptoms (75%), more frequent negative symptoms (87.5%), and more symptoms of disorganization (67.5%). Further, the recovered group differed in their quality of life at follow-up (mean difference=-21.7, *P*<.001) but did not differ in depressive symptoms or general functioning.

#### Characteristics of recovered and non-recovered groups

In a logistical regression analysis we found that recovery was statistically correlated with higher age at baseline assessment (*P*=.009), higher scores on positive symptoms (*P*=.001), lower scores on negative symptoms (*P*<.001), lower scores on depression (*P*=.010), and lower level of aggression (*P*=.012). Other predictors of good recovery were high work performance (*χ*^2^=14.04, *P*<.001) and the ability to live independently (*χ*^2^=4.433, *P*=.035).

Characteristics of non-recovered patients were lower age at intake (*P*<.001), more extra-pyramidal symptoms (*P*<.001), more severe aggressive symptoms (*P*<.001), higher frequency of disorganization symptoms at baseline (*P*=.023), and higher level of family burden at the end of the term (*P*<.001).

### Multidimensional outcome parameters

When patients were evaluated in terms of meeting the criteria for normal scores on seven clinical parameters (negative symptoms, positive symptoms, disorganization, hospitalization in the past two years, suicidality, extra-pyramidal function, and aggression), only five (5.0%) of the 101 patients were found to have normal scores indicating good outcome on all seven parameters. When patients were evaluated in terms of meeting the criteria for normal scores on five social parameters (independent living, interpersonal/social, work/school, quality of life, and GAF), none of the 101 subjects had normal scores indicating good outcome on all five parameters. Eleven patients (10.9%) had good outcome on four of the five parameters. These 11 patients were among the 61 that were classified as recovered according to the CGIS-I. Although the number of parameters on which participants obtained normal social scores was greater among those who recovered (M=2.6) compared to those that did not recover (M=0.80, *P*<.001), there were 29 (47.5%) recovered patients who had fewer than three normal scores on the social parameters. There was a statistically significant relationship between the number of normal social parameters and the number of normal clinical parameters (Spearman *r*=0.337, *P*<.001) indicating a linear relationship.

## DISCUSSION

### Recovery: Overall outcome

The present research aimed at contributing to the literature on schizophrenia outcome, particularly regarding prognostic predictors and level of outcome. The available literature provides some reasonable estimates of recovery rates and details of what factors are associated with long-term outcome.[5,8] The finding of 60% patients with good outcome at 10 years post diagnosis is consistent with what has been previously reported. It is noteworthy that in the present study, 40% of patients who had undergone intensive, comprehensive pharmacological and psychosocial management did not show reasonable recovery. Those who did not recover were younger in age than those who showed recovery on their first hospitalization (predominantly males, mean duration of illness being 10.8 months). These elderly patients presented with severe negative symptoms, severe depression, poor work function and poor independent living status.

What should we be looking for in terms of outcome? Currently, there are no basic minimum criteria to objectively define what is considered a “good outcome.” While it has been repeatedly demonstrated that social outcome is a necessary component in measuring outcome, this criteria is still not used in practice. The number of parameters that should be routinely used in the assessment of recovery needs to be defined. It has been customary to talk about course and outcome together, suggesting the interdependency of these two variables. This perhaps arises from the understanding of a linear relationship between poor illness course leading to poor outcome and good illness course leading to good outcome. But this premise remains uninvestigated. Do patients with schizophrenia running a poor course always develop poor outcome? We do not know the answer yet. At best, illness course can be one factor implicated in determining the level of outcome, but it does not represent the sole determinant. In other words, the outcome of schizophrenia needs to be re-conceptualized and recognized as dependent on both clinical and social parameters.

### Multidimensional outcome

In the present study of long-term outcome, when all criteria are considered together, there are no participants who demonstrate full recovery. Only five patients showed recovery on seven clinical criteria and none showed recovery on all five social criteria. Only 11 patients showed good outcome on four social criteria. Thus, using different criteria of what constitutes recovery leads to very different pictures of outcome. It becomes clear that minimum requisite criteria need to be precisely defined in order to determine the proportion of patients who experience good outcome. Further, clinical recovery on multiple parameters is only seen in 25% of the sample and social recovery in only 26% of the sample. Considering that these two are mutually independent groups, the total recovery remains at 50%. If the criteria are less strict, recovery increases to 45% and 49% in clinical and social recovery, respectively, with considerable overlap of the two groups. Thus, measuring outcome with multiple outcome criteria appears to be satisfactory.

Any measure of outcome should contain an assessment of the factors responsible for complete social integration, with or without continued medication. We therefore examined outcome on 12 different criteria and clustered these parameters into two main groups: clinical and social. Clinical outcome includes positive and negative symptoms and hospitalization. Social outcome includes personal, social, functional, and vocational domains.

#### Clinical outcome

In our sample, the mean positive symptoms’ score was reduced by more than 65% between baseline and endpoint. It is known that positive symptoms respond better to antipsychotic treatment than negative symptoms and the findings in the present study are consistent with this literature. Interestingly, there was a statistically significant difference in the baseline scores of HDRS between the two groups of recovered and non-recovered patients. High scores at baseline correlated with poor recovery at the endpoint. These findings are contrary to what has been found in the literature, that the presence of depressive features predicts a better outcome. It is widely known that affective symptoms in schizophrenia respond better to treatment. It is possible that very high scores suggesting severe depression in schizophrenia may be telling a different story. This, however, is a complex interrelationship.

#### Social outcome

In the literature, social recovery (overall good outcome) is measured by different social and occupational parameters and is found to be present in around 40–60% of individuals. Social recovery has several dimensions, including personal, occupational, vocational, acquisition of interpersonal skills, ability to learn new skills, work, employment, ability to take responsibility, and ability to develop and sustain relationships. The present study attempted to find the number of patients recovering on key social outcome parameters: quality of life, employment, interpersonal social skills and independent living. It was observed here that no patients recovered on all parameters. Only 10% of the patients fulfilled a maximum of four parameters. The largest proportion of patients recovered on only two parameters (25.7%). Social parameters appear to be inter-dependent, and yet, recovery on only one parameter is not associated with recovery on any other parameter. As such, some patients are employed but unable to live independently and others show good quality of life but are still unable to be gainfully employed. In this study it was observed that only 23-25% patients show social recovery on two or three different parameters, far less than what has been previously shown in the literature. This difference is likely due to the fact that most studies have considered only one or two criteria, such as quality of life and global functioning. These studies seem to believe that these two parameters capture the full picture of social recovery, which is seldom true.

### Clinical predictors of recovery

At baseline assessment, levels of clinical symptoms were associated with recovery 10 years later. Specifically, fewer negative symptoms, lower depression scores, and low levels of aggression at baseline predicted recovery. A higher level of positive symptoms at baseline also predicted recovery. This finding is in accordance with previous research suggesting that positive symptoms do have a negative effect on functional capacity and outcome.[27] Recovery was also predicted by a higher age at baseline assessment. Additionally, non-recovery was associated with more extra-pyramidal symptoms and more symptoms of disorganization.

### Social predictors of recovery

In the present study, the two social variables that predicted later recovery were initially high levels of work performance and the ability to live independently at baseline. Overall in this sample, there was not a significant change in the number of patients showing poor work performance or poor interpersonal social functioning from baseline to endpoint, suggesting that treatment programs for schizophrenia require much more of a focus on the social aspects of patients’ lives in addition to the treatment of clinical symptoms.

### Limitations

Although this study has many strengths, it also has some limitations. Primarily, it has a high number of participants lost to follow-up. Losing 49% of the original sample at the follow-up is a considerably high number and some possible causes for this could be the complex social situation, unfulfilled expectation of recovery, lack of incentive, lack of free medication supply, and the clients’ ability to make choices regarding their treatment settings. In the city of Mumbai, where the study was carried out, patients frequently shift between free and private facilities depending on their financial situation and their level of faith in the treatment centre. Migration, the second most significant factor leading to a loss of participants, is always high in metro cities due to changes in opportunities for work.

Although the study design could have been improved, conducting this type of research in naturalistic settings has its own merits as well as limitations. The main strength of the study is that it reflects the pragmatic real-life situation that exists in clinical settings.

## CONCLUSION

Based upon the findings of the present study, clinical information is not sufficient to make an accurate prediction of outcome status. The outcome depends upon several factors, such as the nature of illness, its phenomenology, level of psychosocial stress, neurodevelopmental state, cognitive factors, family pathology, and so on. While it is desirable to achieve remission and recovery on all possible parameters, it is an unmet need in schizophrenia. Outcome needs to be measured on a minimum two groups of parameters, each having at least three or more parameters: clinical outcome (such as psychopathology, side-effects and hospitalization) and social outcome (such as quality of life, global assessment of functioning and social cognition).

An understanding of response, remission, and recovery can be very helpful in direct clinical practice. A major implication of this research is the argument for moving toward a comprehensive assessment of outcome and to plan management accordingly. Bringing social outcome measures to the forefront and into the communities will allow for a more patient-centric approach. It also opens newer vistas for addressing the complex interaction of clinical and social parameters. A change in clinical excellence would be a welcome experience.
